# Lumped-parameter model as a non-invasive tool to assess coronary blood flow in AAOCA patients

**DOI:** 10.1038/s41598-023-44568-8

**Published:** 2023-10-14

**Authors:** Valentina Ceserani, Mauro Lo Rito, Mauro Luca Agnifili, Ariel F. Pascaner, Antonio Rosato, Serena Anglese, Miriam Deamici, Jessica Negri, Chiara Corrado, Francesco Bedogni, Francesco Secchi, Massimo Lombardi, Ferdinando Auricchio, Alessandro Frigiola, Michele Conti

**Affiliations:** 1 Department of Civil Engineering and Architecture, University of Pavia, 20100 Pavia, Italy; 2 Department of Congenital Cardiac Surgery, IRCCS Policlinico San Donato, 20097 San Donato Milanese, Italy; 3 Department of Clinical and Interventional Cardiology, IRCCS Policlinico San Donato, 20097 San Donato Milanese, Italy; 4 3D and Computer Simulation Laboratory, IRCCS Policlinico San Donato, 20097 San Donato Milanese, Italy; 5 Department of Biomedical Sciences for Health, University of Milan, 20122 Milano, Italy; 6 Department of Radiology, IRCCS Policlinico San Donato, 20097 San Donato Milanese, Italy; 7 Multimodality Cardiac Imaging Section, IRCCS Policlinico San Donato, 20097 San Donato Milanese, Italy

**Keywords:** Biomedical engineering, Engineering

## Abstract

Anomalous aortic origin of the coronary artery (AAOCA) is a rare disease associated with sudden cardiac death, usually related to physical effort in young people. Clinical routine tests fail to assess the ischemic risk, calling for novel diagnostic approaches. To this aim, some recent studies propose to assess the coronary blood flow (CBF) in AAOCA by computational simulations but they are limited by the use of data from literature retrieved from normal subjects. To overcome this limitation and obtain a reliable assessment of CBF, we developed a fully patient-specific lumped parameter model based on clinical imaging and in-vivo data retrieved during invasive coronary functional assessment of subjects with AAOCA. In such a way, we can estimate the CBF replicating the two hemodynamic conditions in-vivo analyzed. The model can mimic the effective coronary behavior with high accuracy and could be a valuable tool to quantify CBF in AAOCA. It represents the first step required to move toward a future clinical application with the aim of improving patient care. The study was registered at Clinicaltrial.gov with (ID: NCT05159791, date 2021-12-16).

## Introduction

Anomalous aortic origin of the coronary artery (AAOCA) is a rare congenital disease with several anatomical variants, such as the origin from the opposite sinus of Valsalva^1^. In some cases, an intramural tract may also be present in the most proximal zone, whereby part of the coronary artery (CA) is enclosed within the aortic wall^2^. Although AAOCA is often asymptomatic^3^, it is related to sudden cardiac death (SCD) or ischemic events, usually associated with high-intensity physical activity, especially in young athletes^4,5^.

Risk estimation based on pre-operative tests is critical to AAOCA diagnosis and management because traditional clinical exams, such as stress tests, usually fail to recognize adverse events related to ischemia and SCD^6^. Moreover, the mechanisms linking the anatomical abnormality with the risk of SCD are still unclear.

In this context, computational simulations have contributed to clarifying some mechanical and physiological aspects linked to ischemia since they can simulate extreme stress conditions, which are not traditionally evaluated in diagnostic tests. Previous biomechanical studies using structural finite element analysis^7^ have shown a limited expansion of the anomalous CA under increasing pressure, particularly within the intramural tract^8^. Furthermore, 3D computational fluid dynamics (CFD)^9–11^, and 3D fluid-structure interaction (FSI) simulations^12–14^were performed to assess coronary blood flow (CBF) and coronary perfusion in AAOCA. However, it is worth noting that these hemodynamic investigations were based on the analytically computed coronary resistance based on physiological hypotheses for healthy subjects, probably poorly reliable in AAOCA patients. In addition, the results were not validated due to the lack of in-vivo CBF measurements. Finally, the available patient-specific simulations deal with a small cohort of patients, not attaining general conclusions for the AAOCA population. Therefore we aimed to develop a patient-specific lumped-parameter model (LPM) fed by anatomical, hemodynamic, and functional data retrieved from AAOCA patients that could accurately estimate CBF at two different conditions. We validate the model accuracy in replicating in-vivo conditions by comparing the results with in-vivo measurements.

## Methods

This study was approved by the IRCCS Policlinico San Raffaele ethical committee (record number 19/int/2021, approved 09/06/2021). All patients enrolled in the study at IRCCS Policlinico San Donato gave written informed consent. All methods were carried out in accordance with relevant guidelines and regulations.

In particular, the present work includes data retrieved from 19 AAOCA patients, recruited for the NECESSARY study (GR-2019-12369116, Italian Ministry of Health) between November 2021 and May 2023 (ClinicalTrials.gov Identifier: NCT05159791). The study population consists of patients with AAOCA, subjects with other congenital cardiac abnormalities or contraindications to performing the required diagnostic tests were excluded. All patients recruited did not present atherosclerotic plaque and calcification or diagnosis of severe stenosis.

**Figure 1:**
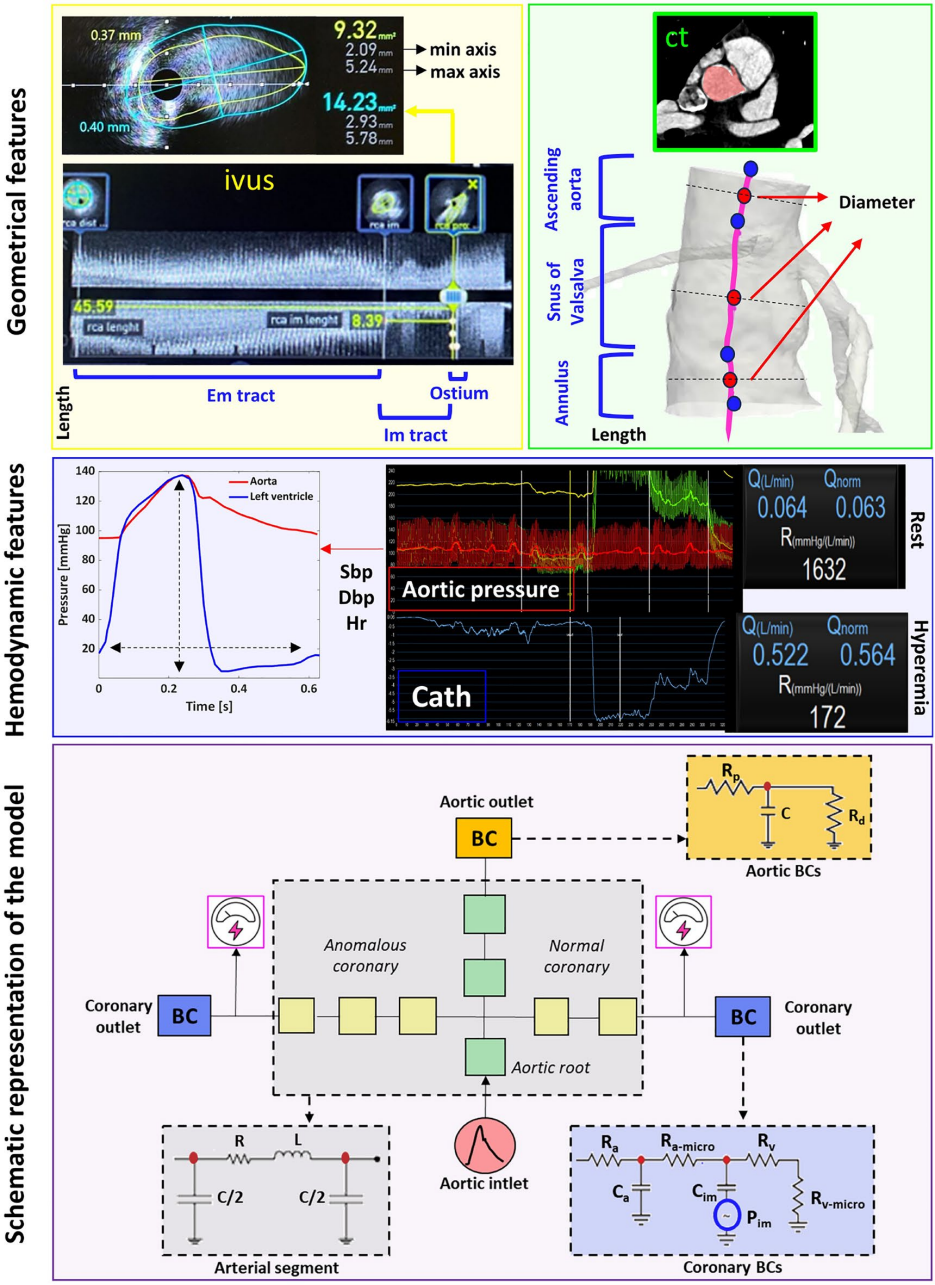
Schematic representation of the lumped-parameter model and input data used to calibrate it. On the top, IVUS registration was illustrated in the yellow rectangle: a slice was selected to extract the geometrical measurements related to the more proximal tract (OSTIUM) of a right CA. In the green rectangle, the points selected along the centerline of the 3D model of the aortic root were shown; they were used to measure aortic geometrical parameters. The 3D model was reconstructed through the segmentation of CT. In the middle, signals recorded during the coronary functional assessment were shown in the blue rectangle. For both rest and hyperemic conditions CBF (Q) and CA resistance (R) were assessed. Aortic pressure wave signal was used to extract patient values to rescale both aortic and left ventricular pressure waves taken from literature^14^. On the bottom, the purple rectangle enclosed a schematic representation of the lumped-parameter model. Each arterial segment was represented by the circuit highlighted in gray, aortic BCs were defined by the Windkessel circuit colored in orange, and the BC of the CAs were defined in the blue dashed rectangle.

The study involved the collection of clinical data and coronary anomaly classification. Details regarding the acquisition, post-processing, and use of such data are described in the following sections, and a schematic representation is also shown in Fig. 1.

### Clinical data acquisition

Computed tomography (CT) scans were performed on a dual-source CT system (Somatom Flash; Siemens Medical Solutions, Forchheim, Germany). CT parameters were 0.6 mm slice thickness, 0.3 mm increment, kernel Bv38, and Admire with strength 2. For patients that had performed the CT in other centers (n$=$16), a maximum slice thickness of 0.6 mm was required.

Cardiac magnetic resonance imaging (MRI) examinations were performed with a 1.5 T unit (Magnetom AERA, Siemens Medical Solutions, Forchheim, Germany) with 45 mT/m gradient power, using twelve channel surface phased-array coil placed over the thorax and with the patient in supine position. Image acquisition was gated by the electrocardiogram (ECG) signal and respiration control (breath-holding) to produce a CINE sequence throughout all the systole and diastole and to avoid cardiac and respiratory artifacts. An MRI study included a complete set of short-axis (from base to apex) CINE images, using an ECG-triggered steady-state free precession pulse sequence acquired with the following technical parameters: TR/TE 4.0/1.5 ms; FA 80°; slice thickness 8 mm; time resolution 45 ms; mean acquisition time 14±4 s.

In addition, the coronary anomalies were investiagated with intravascular unltrasound (IVUS) and with a coronary functional evaluation protocol. For the IVUS we adopted the OptiCross intravascular ultrasound with a 40MHz rotational transducer (Boston Scientific; Marlborough, Massachusetts, USA). Pullbacks were performed with a motorized system at a constant speed of 1 mm/s. Images were acquired at 30 frames/s in digital format, and stored into the PACS system. The coronary functional evaluation was performed with Coroventis CoroFlow Cardiovascular System (Abbott; Chicago, Illinois, USA), which is an advanced platform capable of calculating hemodynamic measurements in both epicardial vessels and coronary microvasculature. Absolute CBF and coronary resistances, expressed in L/min and mmHg/(L/min) respectively, were measured by continuous thermodilution technique as protocols available in literature^15,16^. The measurements were done at rest and at maximum hyperemia. Imaging data were saved in DICOM format in order to be processed. Other information recorded during the coronary functional assessment were saved in “.csv” format.

### Clinical data post-processing

CT images were semi-automatically segmented to create a 3D patient-specific reconstruction of the aortic root and the CAs using Mimics software (Materialise HQ, Leuven, Belgium). Lumen centerlines were computed using Vmtk 1.4.0 software (The Vascular Modeling Toolkit, Orobix srl, Bergamo, Italy) and centerline-based geometrical informations were obtained using Paraview 5.10.0 (Kitware Inc, New York, USA.). Minimum and maximum diameters of aortic cross-sections were extracted at three anatomical sites: annulus, sinus of Valsalva at the sinotubular junction, and proximal ascending aorta. The distance between each pair of consecutive section centroids was also measured , as shown in the green rectangle of Fig. 1.

MRI segmentation was performed using MEDIS QMass 7.6 (Medis medical imaging systems bv, Leiden, The Netherlands). For the segmentation of cardiac images, in each session, the reader manually traced the epicardial and endocardial contour of the entire heart on the short axis plane, both in the tele-diastolic and tele-systolic phases. Then, the software automatically calculated the end-diastolic volume (EDV), end-systolic volume (ESV), stroke volume (SV), ejection fraction (EF), and cardiac mass values. SV was calculated as the difference between EDV and ESV. For patients that did not undergo an MRI examination due to specific associated risk (n$=$2), the SV was taken from literature based on sex and age^17^.

IVUS segmentation was manually performed by an expert clinician (MLR, MLA) using POLARIS Multi-Modality Guidance System software (Boston Scientific; Marlborough, Massachusetts, USA), allowing real-time visualization of IVUS frames and evaluation of the maximum and minimum axes of the selected region, the wall thickness, and the length of the coronary tract between two consecutive selected frames. An example was illustrated in the yellow rectangle of Fig. 1.

The pressure wave recorded during the coronary functional assessment was synchronized with the ECG signal, and ten consecutive cardiac cycles were selected to compute mean maximum (i.e., systolic) and minimum (i.e., diastolic) pressure. The time between consecutive R peaks in the ECG signal was also measured to define each patient’s mean heart rate (HR) during rest and hyperemic CBF in-vivo measurements. Signals were processed using Matlab software (The MathWorks Inc., Natick, USA).

### Model definition

As shown in Fig.1, we adopted a simplified Westerhof circuit^18^ to define each vessel of our domain of interest. We used three circuit components for the aortic root, considering each segment as a perfect cylinder with a circular cross-section. In turn, each CA was assumed to be elliptical, as it better describes the anatomy of anomalous CAs^5^. We used two and three compartments to represent the normal and anomalous CA, respectively.

The electrical components of each circuit depended on the dimensions of the arterial segment (length, cross-section radius, area, and wall thickness), as well as the mechanical properties of the wall and the blood. The model was implemented by Matlab toolbox Simulink v2021a (The MathWorks Inc., Natick, USA).

### Model calibration

#### Geometrical features

Three consecutive aortic segments were analyzed from CT images, as described in the previous section, to define the aortic root. The average diameter of each tract and its respective length were measured. Wall thickness is set to 3 mm according to the literature^19,20^.

Geometrical information regarding the CAs was measured from IVUS as reported in the previous section; the maximum and minimum axes were used to compute the radius and area of the elliptic cross section^21^ considering the frames selected by the clinician in the proximal and distal tract of each CA. For patients that did not undergo IVUS (n$=$2), geometrical measurements of the CAs were extracted from CT images, and wall thickness was set as 0.3 mm as the average population collected values.

#### Mechanical properties

The blood was assumed to be a Newtonian fluid, with a 1.06 g/cm$^3$ density and dynamic viscosity of 0.04 P^14^. A linear elastic material with a Young’s modulus of 1.5 MPa is used to describe the mechanical behavior of the arterial wall^14^.

#### Aortic inlet boundary condition (BC)

The aortic pressure is used as an inlet BC as shown in Fig.1. In particular, the pressure waveform proposed by Jiang et al.^14^ is rescaled in amplitude and time to match the aortic pressure wave recorded during the catheterization exam. In particular, the left ventricular pressure crossed the aorta wave shortly after the start of systole^14^, as shown in Fig. 1. Such a curve and the related Fourier series decomposition are used as data input for the voltage source implemented in Simulink to simulate thirty heart beats.

#### CAs outlet BC

The CAs outlet is defined by a 6-elements circuit, according to Kim et al.^22^. As shown in Fig. 1, such a circuit is composed of four resistors to describe arterial and venous circulation (R$_a$, R$_v$) and microcirculation (R$_{a-micro}$, R$_{v-micro}$), two capacitors to define arterial (C$_a$) and myocardial compliance (C$_{im}$), and a voltage generator to describe the intramyocardial pressure (P$_{im}$). In-vivo measured coronary resistance is used as the total resistance of each outlet and is split among the resistive components following the percentage proposed by Sankaran et al., 32$\%$, 52$\%$, and 16$\%$ for R$_a$, R$_{a-micro}$, and the series of R$_v$ and R$_{v-micro}$, respectively^22,23^. In-vivo measurements are available for rest and hyperemic hemodynamic conditions. The left ventricular pressure (LVP) waveform is scaled based on patient-specific data, whereas the right one is approximated as 20$\%$ of LVP^23^. The values of the two capacitors (C$_a$ and C$_{im}$) are taken from the literature^14^.

#### Aortic outlet BC

The aortic outlet is modeled with a Windkessel circuit (Fig.1) according to Westerhof et al.^24^. The total aortic resistance is evaluated according to Sankaran et al.^23^ using the patient-specific cardiac output (CO) computed as the product between SV and HR, and the patient-specific mean arterial pressure. The total resistance is distributed among the circuit parameters allocating 91$\%$ and 9$\%$ to R$_d$ and R$_p$, respectively. The compliance value (C) is taken from the literature and set to 0.001 cm$^5$/dyn^14^.

### Simulation setup

The measurements were converted into cgs units for consistency with the adopted model^18^. Considering each vessel as an electric circuit, as shown in Fig. 1, the respective differential equations^25^are solved. In particular, the implementation in Simulink was performed, increasing the actual frequency by a factor of 1000^18^ to lower the required computational time and reduce the values of capacitance and inductance. Thus, all physical quantities are appropriately scaled in order to convert hemodynamic features into electrical ones.^18^. Thirty heartbeats are simulated. Since rest and maximum hyperemia are investigated during the catheterization exam, the coronary functional assessment by the numerical model is also performed at these two hemodynamic conditions.

### Statistical analysis

Due to the small sample size, both measured in-vivo CBF and computed in-silico CBF were expressed as median value and interquartile range (IQR). Accordingly, non-parametric statistical tests were performed. Wilcoxon signed-rank test was used to analyze differences between in-vivo and in-silico CBF, setting $\alpha$ = 5$\%$ and considering a two-tailed test; these differences were considered significant for *p*-value < 0.025. Spearman’s correlation coefficient was computed to assess the correlation between the two measurements, and Bland-Altman analysis was also performed. Furthermore, relative differences between in-silico and in-vivo CBF were evaluated in order to study their correlation with input data such as coronary resistance, stroke volume, systolic pressure, and CAs size.

Finally, whereas the coronary resistances presented a strong correlation with in-vivo data, due to the procedure of measurements, an uncertainty quantification analysis was performed considering a single patient. In particular, a normal distribution of right and left coronary resistances was hypothesized with a mean value equal to the in-vivo measurements and a standard deviation progressively higher. 100 samples were extracted randomly, thus simulations at rest were performed. The impact of the input variability on the results was assessed.

## Results

The study population includes 19 AAOCA patients, 15 men and 4 women with a median age of 50(20). Most of the subjects (n$=$15) had an anomalous origin of right CA, while a minority (n$=$4) had anomalous left CA. Values of in-silico and in-vivo CBF for all patients are presented in Table 1.

**Table 1:** In-silico and in-vivo CBF.

ID	AN	CBF at rest (ml/s)						CBF at Hyperemia (ml/s)					
		LCA			RCA			LCA			RCA		
		In-silico	In-vivo	Rel diff	In-silico	In-vivo	Rel diff	In-silico	In-vivo	Rel diff	In-silico	In-vivo	Rel diff
1	RCA	1.65	1.72	−0.04	0.76	0.75	0.01	7.54	7.75	−0.03	6.84	6.25	0.09
2	LCA	1.03	1.02	0.01	1.72	1.73	−0.01	5.24	5.05	−0.41	5.30	5.53	−0.04
3	RCA	0.91	0.97	−0.06	2.66	2.67	0.00	3.50	3.47	0.01	4.66	4.77	−0.02
4	RCA	1.68	1.75	−0.04	1.06	1.03	0.02	7.41	7.23	0.02	4.37	3.53	0.24
5	RCA	2.06	2.23	−0.08	1.04	1.23	−0.16	6.02	6.20	−0.03	3.21	4.03	−0.20
6	RCA	1.60	1.73	−0.07	1.23	1.25	−0.01	8.21	7.25	0.13	4.67	4.58	0.02
7	LCA	1.04	1.13	−0.08	1.79	1.85	−0.03	5.90	5.65	0.04	5.29	4.13	0.28
8	LCA	1.05	1.07	−0.01	1.37	1.33	0.03	9.89	8.70	0.14	5.73	5.25	0.09
9	RCA	2.82	3.22	−0.12	0.871	0.87	0.00	7.25	6.73	0.08	3.93	2.83	0.39
10	RCA	1.25	1.37	−0.09	0.79	0.83	−0.05	2.98	3.00	−0.01	2.76	2.45	0.13
11	RCA	1.21	1.18	0.02	0.43	0.43	0.00	5.83	5.62	0.04	2.45	2.33	0.05
12	RCA	9.76	10.32	−0.05	0.96	1.02	−0.06	13.84	14.22	−0.03	3.30	2.98	0.11
13	RCA	1.78	1.48	0.20	1.04	1.13	−0.08	4.16	2.85	0.46	2.25	2.32	−0.08
14	RCA	1.13	1.18	−0.04	0.87	0.90	−0.03	5.61	5.72	−0.02	3.48	3.53	−0.02
15	RCA	2.45	2.50	−0.02	2.13	2.03	0.05	3.62	3.62	0.00	3.02	2.83	0.07
16	LCA	3.59	3.43	0.05	4.11	3.98	0.03	7.55	7.13	0.06	8.33	7.45	0.12
17	RCA	1.77	1.73	0.02	1.96	2.08	−0.06	5.51	5.02	0.10	4.43	3.03	0.46
18	LCA	1.33	1.78	−0.25	3.67	3.65	0.01	6.79	6.32	0.07	7.31	7.07	0.03
19	RCA	0.71	0.83	−0.15	0.74	0.97	−0.23	2.08	2.57	−0.19	1.97	2.50	−0.21
25% percentile		1.05	1.13	−0.08	0.87	0.90	−0.06	4.16	3.62	−0.02	3.02	2.83	−0.02
Median		**1.60**	**1.72**	**−0.04**	**1.06**	**1.23**	**−0.01**	**5.90**	**5.72**	**0.04**	**4.37**	**3.53**	**0.07**
75% Percentile		2.06	2.23	−0.01	1.96	2.03	0.01	7.54	7.23	0.08	5.30	5.25	0.13

In-silico computed CBF, in-vivo measured CBF , and respective relative differences (rel diff) are reported for all patients considered in both hemodynamic conditions analyzed (rest and hyperemia). For each patient the anomalous CA is highlighted: left CA (LCA) /right CA (RCA). Median, 25$\%$ percentile, and 75$\%$ percentile of CBF value are also reported

The simulations resulted in median values of CBF at rest of 1.60 (1.01) ml/s and 1.06 (1.09) ml/s, for the left and right CA, respectively. Due to the vasodilatation, hyperemic left and right CBF rise to 5.90 (2.28) ml/s and 4.37 (3.38) ml/s, respectively.

Considering the invasive measurements, the median of the in-vivo measured CBF 1.72(1.10) ml/s and 1.23(1.13) ml/s, for the left and right CA, respectively. Due to the vasodilatation, hyperemic left and right CBF rise to 5.72(3.61) ml/s and 3.53(2.42) ml/s, respectively.

**Figure 2:**
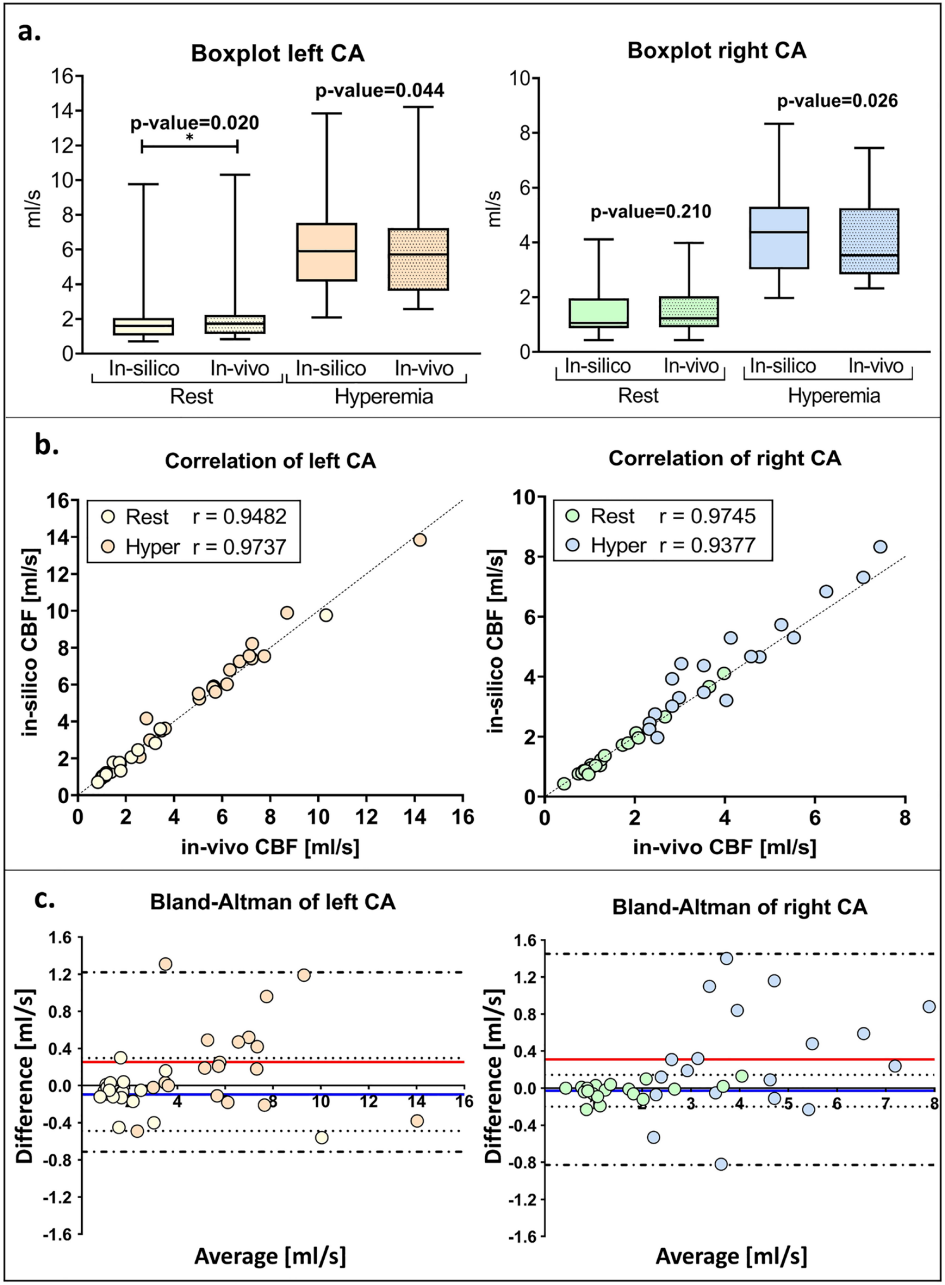
Results of the model and comparison with in-vivo data. (**a**) In-silico computed CBF and in-vivo measured CBF are shown in the boxplots; only significant differences are highlighted (*). (**b**) Scatter plots and correlation analysis are shown. The results related to the left CA are presented on the left, and those corresponding to the right CA are reported on the right. Left CA results are represented in yellow and orange for rest and hyperemic conditions, respectively. Right CA results are presented in green and blue for rest and hyperemic conditions, respectively. (**c**) Bland-Altman plot analysis was shown for both left and right CA. Difference between in-silico and in-vivo CBF were reported on y-axis, the average was reported on the x-axis. Horizontal dashed lines represented the the 95$\%$ limits of agreement of rest (docs) and hyperemic (doc and line) condition. The blue and red lines represented the average discrepancy between in-silico and in-vivo measurements for rest and hyperemia, respectively.

Comparison and correlation analysis performed between in-silico and in-vivo measurements were shown in Fig. 2. In particular, a statistically significant difference (*p* = 0.020) was shown only for the left CA at rest, whereas the rest of the comparisons showed no significant differences (see Fig. 2A). More in detail, Spearman’s correlation evaluation and Bland-Altman analysis suggested that the model tends to underestimate in-vivo CBF when the rest condition was simulated, presenting a mean negative bias of −0.10 ml/s and −0.03 ml/s for the left and right CAs, respectively (blu line in Fig. 2C). A better assessment of right CBF at rest was also confirmed by the Spearman’s coefficient value that presented a value of 0.9745, higher than 0.9482 of the left CA, as reported in Fig. 2B. Considering hyperemic conditions, no significant differences were highlighted by the Wilcoxon signed-rank test (see Fig. 2A). Nevertheless, the accuracy of the model decreased with respect to the previous condition analyzed for the right CA. Indeed, the model tends to overestimate the real CBF showing a positive bias of 0.31 ml/s, with a limit of agreement between −0.83 ml/s and 1.45ml/s, and a Spearman’s correlation coefficient of 0.9377. Whereas, the left CA showed a bias of 0.25 ml/s (line red in Fig. 2C) with a limit of agreement within −0.71 ml/s and 1.22 ml/s; the Spearman’s correlation coefficient reached the value of 0.9737. The Bland-Altman analysis highlighted a trend by which higher CBF values corresponded to higher bias, thus higher average discrepancy between in-silico simulated value and in-vivo measurements.

Relative differences between in-silico and in-vivo data showed a small negative median value for both sides at the rest (left −0.04; right −0.01), whereas little higher positive values were highlighted considering the hyperemia (left 0.04; right 0.07) as shown in Table 1. Correlation analysis performed with input parameters did not show any significant results, details were reported in supplementary materials (SM). The input data used was also reported in SM: coronary geometrical measurements were reported in Table S2; in-vivo measured total coronary peripheral resistances for both hemodynamic conditions studied are reported in Table S3; details regarding the inlet pressure wave are reported in Table S4.

Uncertainty quantification analysis highlighted that the model accuracy is lower the more the coronary resistance value deviates from that in-vivo measured, for both the left and right side. More details were reported in Supplementary Material.

Regarding in-vivo CBF measurements it was possible to compute the flow split between the CAs. Considering our population the median flow split at rest was 57(20)$\%$ and 43(20)$\%$ between left and right CAs respectively. Stratifying the population by anomalous side, patients with right anomalous CA showed a flow split of 60(17)$\%$ and 40(17)$\%$, while patients with left anomalous CA had a flow split of 38(10)$\%$ and 62(10)$\%$ for left and right CAs respectively. Considering hyperemic measurements, general population flow split was 58(11)$\%$ and 42(11)$\%$, patients with anomalous right CA showed values of 61(13)$\%$ and 39(13)$\%$, while values of patients with anomalous left CA were 49(13)$\%$ and 51(13)$\%$ for left and right CAs respectively.

## Discussion

We have developed a patient-specific LPM to compute CBF in patients with AAOCA. Indeed, due to the poor understanding of the mechanisms behind SCD in this kind of patients, it is crucial to assess ischemic risk through pre-operative tests in the management of this rare disease. For this reason it was essential to have a tool that could evaluate CBF under increasing levels of effort and determine the oxygen supply to the myocardium. By performing in-silico simulations, we have successfully recreated the hemodynamic conditions that were previously examined through invasive procedures in-vivo. Firstly, our findings confirm that we can create a fully patient-specific LPM to assess the CBF in AAOCA using the clinical data of patients. Secondly, the presented patient-specific LPM demonstrated the ability to simulate different hemodynamic conditions, allowing to explore different scenarios and deepening the complex pathogenetic mechanisms of AAOCA. Finally, the LPM we presented accurately replicates the hemodynamic conditions previously analyzed in-vivo.

Previous computational models implemented to analyze the hemodynamics in AAOCA patients relied on assumptions derived from the literature regarding the physiological coronary circulation of healthy patients^23,26^. Although these models incorporated patient-specific data to analytically compute coronary peripheral resistances, direct validation of the analytical results was missing^9–11,14^. In our study, we measured coronary peripheral resistances in-vivo, enabling us to simulate AAOCA CBF in a fully patient-specific manner. Unfortunately, our value could not be directly compared to the values presented in other studies due to non-reported^14^ or related to a different number of coronary outlets considered^11,16,22,23^.

In particular, during the invasive procedure, coronary resistances were directly measured in two different hemodynamic conditions: the resting state representing the normal physiological condition, and the epicardial coronary response to effort induced by hyperemia.This coronary invasive functional assessment did not involve drugs. Rest and hyperemic conditions were evaluated through an infusion of saline solution by thermodilution technique. No coronary indexes were measured and only absolute values of CBF and coronary resistances were computed. For these reasons, with respect to previous other works, in which computational 3D simulations were used to study wall shear stress distributions^11^ or instantaneous wave-free ratio (iFR) index^14^ in AAOCA patients, our interest lies in simulating the CBF and quantifying it at different hemodynamic conditions in a fully patient-specific, fast, and replicable way. Our LPM can simulate the CBF of a single patient within seconds, making it a promising candidate to be introduced into clinical practice as a supportive instrument for clinical, surgical, and therapeutic decision-making. Furthermore, to the best of our knowledge, there are no studies reporting in-vivo measurements of CBF in patients with AAOCA. Thus, our measurements enable us to both test the accuracy of the model in replicating specific hemodynamic conditions and analyze the data to study the impact of pathology on the CBF compared to healthy subjects. Analyzing the accuracy of the model under both simulated hemodynamic conditions, our numerical tool results exhibited a strong correlation with in-vivo measurements, even if we observed higher correlation coefficients at rest compared to hyperemia. Also the Bland-Altman analysis showed a bias of 0.03 ml/s at rest which reached the highest value of of 0.3 ml/s in hyperemia, corresponding to a percentage error of 4$\%$ and 7$\%$ respectively. An error of this magnitude is in any case clinically negligible. This confirms that our LPM is able to estimate CBF in AAOCA patients with high accuracy.

Analyzing the average CBF values of the included patients and comparing them with data from the literature, notable differences in coronary perfusion between healthy individuals and AAOCA subjects became evident. In particular, the normal flow split between the left CA and right CA of 70$\%$ and 30$\%$, respectively, was not observed in AAOCA patients. The subjects analyzed in this study showed an average flow split of 57$\%$ and 43$\%$ for left CA and right CA, respectively. Stratifying the population by anomalous side, subjects with anomalous right CA showed the same distribution, while subjects with anomalous left CA exhibited an opposite flow split, as reported in the previous section. These results could significantly impact future works highlighting a different physiological behavior of AAOCA in comparison with healthy patients.

Although the proposed model can reproduce the considered hemodynamic conditions with excellent results, since our aim is to study CBF at different degrees of effort, the model necessitates the consideration of multiple factors. Currently, we have only accounted for coronary peripheral resistance reduction, thus we were not able to analyze the impact of increasing pressure and HR on CBF assessment. Future work might incorporate these variations in order to simulate different degrees of effort in a more realistic way. In addition, in future work, we plan to progressively replace invasive data with non-invasive data developing an algorithm to evaluate coronary resistances based on clinical data retrieved from AAOCA subjects and validating it. In this context, it could be also interesting to consider other invasive indexes able to describe and assess the coronary microvascular state^27^. Another limitation of the presented model is that it is not a closed loop: the venous system is represented by a zero pressure status. More sophisticated and complex models could also consider pulmonary circulation and include the respiratory process and gas exchanges in the simulations. Additionally, in this step the model considered the blood as a Newtonian fluid, and any advanced hemodynamic parameters were evaluated. Even if AAOCA pathological behavior did not induce plaque development and our patients did not present any calcification or stenosis, more accurate and realistic simulations considering the non-Newtonian rheology behavior of blood and its effect on coronary biomechanics could be interesting^28^; with this aim, a different approach could be used. Nevertheless, for the scope of this work, we chose to keep the model as simple as possible, in order to obtain accurate and fast results that could potentially be applied in the clinical routine. With this aim, a key aspect to consider is related to verification, validation, and uncertainty quantification (UQ) processes. In this first developing step, our aim has been to define a method able to quickly and accurately assess the same conditions analyzed in-vivo, so we performed a verification of the model. We performed also a UQ considering the impact of coronary resistance variation on CBF evaluation but a deeper analysis was needed. In a future step, when the model will be calibrated using only non-invasive data to provide a CBF, validation will be fundamental in order to define a patient-specific reliable and accurate model. The impact of the variability of the input data on simulated CBF, especially that of coronary resistance values, will prove the robustness of the method.

## Conclusion

The proposed LPM represents a fast analytical tool that accurately estimates CBF at rest and hyperemia, replicating the AAOCA complex pathogenetic mechanism. Our results suggest that the described method represents the first step towards a non-invasive patient-specific tool to obtain reliable CBF evaluation in AAOCA, allowing to replicate conditions that cannot be reproduced during catheterization or clinical exams.

### Supplementary Information

Supplementary Information.

## Data Availability

Data analyzed during the current study and needed for the replicability of the results are available in the supplementary materials.
