# Supplementary material for: Lumped-parameter model as a non-invasive tool to assess coronary blood flow in AAOCA patients
Source: Sci Rep. 2023 Oct 14;13:17448. doi: 10.1038/s41598-023-44568-8 (PMC10576762; doi:10.1038/s41598-023-44568-8)
Supplement: Supplementary file 1 — Supplementary Information. [file 41598_2023_44568_MOESM1_ESM.pdf]

Lumped-parameter model as a non-invasive tool to assess coronary blood flow in AAOCA patients

Valentina Ceserani, Mauro Lo Rito, Mauro L. Agnifili, Ariel F. Pascaner, Antonio Rosato, Serena Anglese, Miriam De Amici, Jessica Negri, Chiara Corrado, Francesco Bedogni, Francesco Secchi, Massimo Lombardi, Ferdinando Auricchio, Alessandro Frigiola, and Michele Conti.

Supplementary materials

Correlation analysis: relative differences between in-silico and in-vivo data vs. input parameters.

Simulation at rest and hyperemia was performed in order to assess the coronary blood flow (CBF) in 19 AAOCA patients. Relative differences between in-silico and in-vivo data were computed and reported in Table 1 of the main manuscript. Considering the rest results, the correlation between relative differences and input data was analyzed computing Spearman's coefficient r and performing linear regression analysis. The results were shown in Figure 1S. Input data considered into the analysis were: proximal coronary cross section area evaluated using minimum and maximum axis (Table S2) , coronary resistance (Table S3), stroke volume (Table S4), and systolic blood pressure (Table S4). All comparisons done showed no significant correlation, Spearman's correlation coefficient showed always values < 0.5. Also linear regression did not allow to do any consideration about a possible linear relation between the variables considered. No interesting assumptions could be allowed. Input data considered were similar for every patient considered, they seemed to have no impact on the accuracy of the model following a linear relation. For each analysis a linear regression equation was evaluated but p-value was still high.

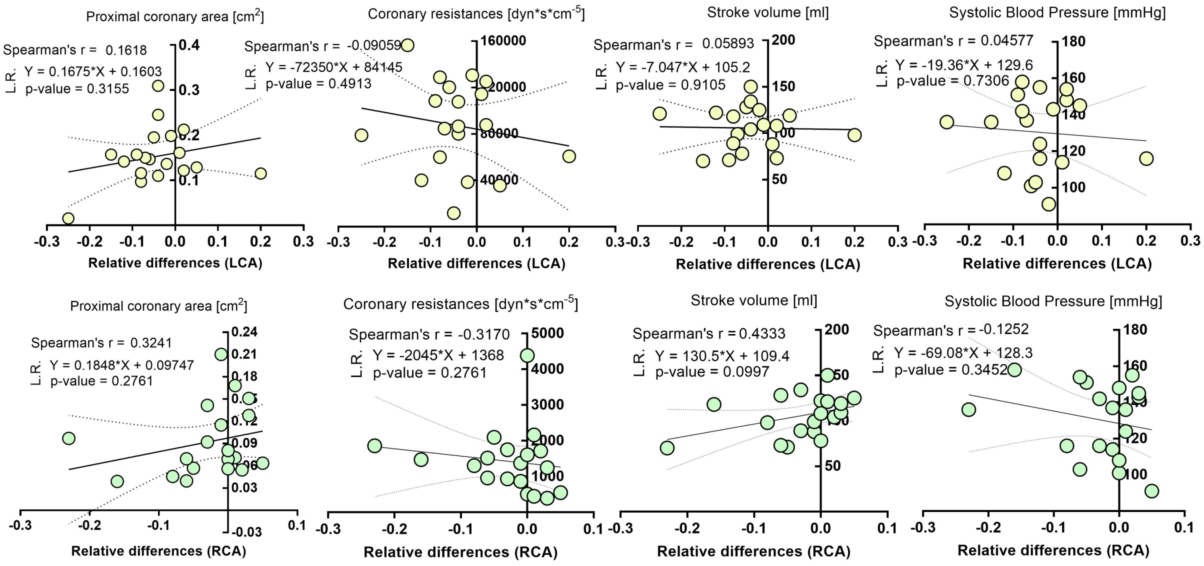

Figure S1. **Correlation analysis between CBF relative differences and input data.** For each relation, details about the results of the test performed were reported: Spearman's correlation coefficient r, equation of regression line, and related p-value. Plot related to left CA and right CA were shown on the top and on the bottom, respectively. Starting from the left side,the results of the correlation analysis between the CBF relative differences and proximal coronary artery area, coronary resistance, stroke volume, and systolic pressure were illustrated.

Uncertainty quantification

Considering the way in which CBF and coronary resistances were computed in-vivo, we know that their values are strongly correlated, so the good accuracy resulting from our analysis suggests that the model is able to replicate the condition studied in-vivo. Thus we have just verified the goodness of the model, but for a realistic validation and uncertainty quantification(UQ) analysis we might test the model with other input data, for example with coronary resistances analytically computed. Indeed, it will be one of the main future developments of the presented model. For this reason, at this step, the UQ analysis could be an interesting assessment of the performance of the model.
Considering a single patient, we have hypothesized a normal distribution of coronary resistance value with a mean equal to the coronary resistance measured in-vivo and a standard deviation progressively higher, equal to 1%, 5%, 10% and 20% of the

mean value. We run the simulation with a random extraction from each distribution of 100 samples, so with a random value of both right and left coronary resistances. We analyzed the impact of the coronary resistance variation on CBF considering the relative differences between in-silico and in-vivo values, in order to assess the error of the model. The relation between the values of the two sides was missing, it would be in depth analyzed. At this moment the results showed that the model accuracy is lower the more the coronary resistance value deviates from that measured in vivo, for both left and right side. In particular, the right side (mostly anomalous) showed higher errors. These considerations suggest that the CBF is strongly related to the coronary resistance value and for a reliable simulation it will be fundamental to assess in the most correct possible way the coronary resistances, especially for the anomalous CAs. The Figure 2S shows the boxplot of the coronary resistances value of 100 samples for each hypothesized variation and related relative differences between in-silico and in-vivo CBF. Right and left results were presented separately. The results showed that when we considered a bigger variation the model resulted in a less accurate simulation of the in-vivo value. In the worst case, when it was allowed a variation of 20% for the coronary resistances the most results fell between a relative error of -0.1120 (25th percentile) and 0.1523 (75th percentile) for the left CA , and of -0.1010 (25th percentile) and 0.2223 (75th percentile) for the right one. Even if the average error is still quite low, in some cases the model assesses the CBF in a completely wrong way, reaching an error of 2.00 (such as 200%). This confirmed that the model results were strongly related to the coronary BCs and a precise assessment of the coronary resistance was needed for a good CBF simulation, especially in AAOCA patients.

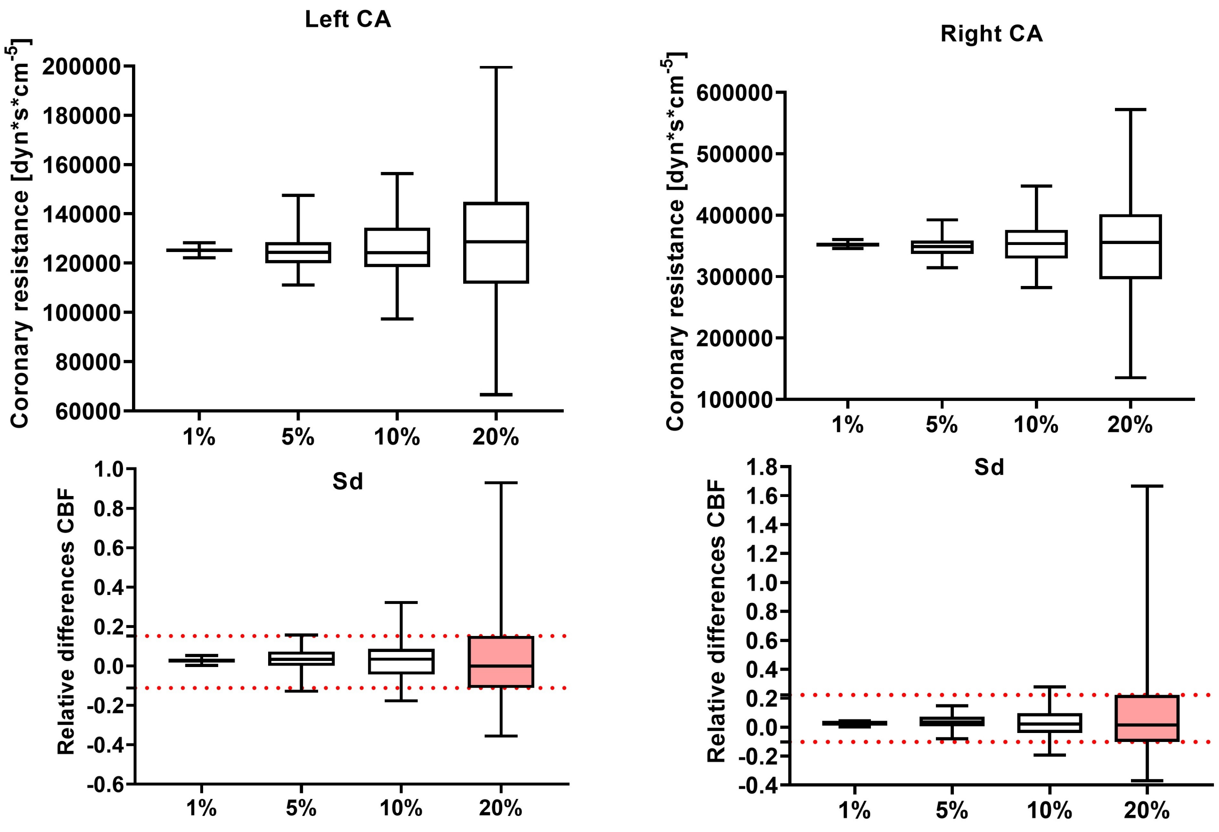

Figure S2. **UQ analysis: coronary resistance variations and impact on the CBF relative differences.** On the top were illustrated the boxplot of 100 samples of coronary resistance values randomly extracted from a normal distribution with standard deviation increasingly higher, from 1% to 20%. On the bottom, relative differences between in-silico and in-vivo CBF were presented for each case of variation analyzed. The 25th and 75th percentiles of the worst cases were highlighted in red. Results of the left CA were reported on the left and of the right CA on the right side of the figure.

Tables

	Aortic root 1		Aortic root 2		Aortic root 3	
**ID**	**D**	**Z**	**D**	**Z**	**D**	**Z**
1	2.67	0.46	2.95	1.06	2.94	1.45
2	2.64	0.61	2.95	1.67	2.65	1.20
3	2.30	0.57	2.31	0.66	2.51	0.85
4	2.71	0.64	3.25	1.07	3.97	1.33
5	2.21	0.36	0.27	0.96	2.51	1.31
6	2.16	0.60	2.49	0.99	2.7	0.52
7	2.22	0.82	3.20	1.49	2.80	1.07
8	2.22	0.51	2.30	1.45	2.53	1.00
9	2.56	0.55	2.48	0.88	2.32	0.91
10	2.11	0.61	2.36	1.04	0.27	0.54
11	2.77	0.56	2.94	1.16	3.32	0.99
12	2.02	0.49	2.88	1.80	2.20	0.58
13	2.36	0.61	2.55	1.26	2.68	0.86
14	3.00	0.63	4.09	2.60	3.30	0.70
15	2.57	0.51	3.40	2.14	3.30	0.82
16	2.70	0.84	3.19	2.05	3.20	0.60
17	2.60	0.38	3.19	2.41	2.70	0.93
18	2.80	0.62	3.39	2.20	2.90	0.72
19	0.23	0.50	3.60	3.00	3.70	1.00

Table S1. Aortic root. Geometrical measurements regarding three consecutive tracts of the aortic root are shown. In particular, *Aortic root 1* corresponds to Annulus, *Aortic root 2* represents the region between annulus and sinotubular junctions, and *Aortic root 3* represents the portion of ascending aorta considered into the model. For each segment, diameter (D) and length (Z) are reported in *cm*. All data were retrieved from CT images.

	Anomalous coronary artery										Normal coronary artery							
	Ostio			IM tract			EM tract				Proximal			Distal				
ID	Max D	Min D	Length	Max D	Min D	Length	Max D	Min D	Length	Source	Max D	Min D	Length	Max D	Min D	Length	Source	H
1	0.55	0.16	1.21	0.40	0.24	0.84	0.38	0.36	3.72	IVUS	0.66	0.60	0.88	0.50	0.44	0.88	IVUS	0.04
2	0.47	0.43	0.01	0.30	0.27	4.18	0.41	0.39	2.75	IVUS	0.58	0.46	1.62	0.49	0.43	1.62	IVUS	0.03
3	0.48	0.19	0.01	0.40	0.26	1.23	0.32	0.30	2.10	IVUS	0.46	0.40	2.22	0.32	0.31	2.22	IVUS	0.03
4	0.61	0.11	0.08	0.35	0.20	0.79	0.38	0.35	2.80	CT	0.38	0.37	0.87	0.39	0.36	0.87	CT	0.03
5	0.34	0.15	0.10	0.18	0.18	1.31	0.32	0.24	1.65	CT	0.43	0.29	1.50	0.50	0.26	1.50	CT	0.03
6	0.65	0.23	0.32	0.54	0.19	0.41	0.40	0.39	2.87	IVUS	0.47	0.41	0.69	0.47	0.38	0.69	CT	0.03
7	0.39	0.38	0.01	0.39	0.32	3.15	0.31	0.27	2.62	IVUS	0.38	0.31	4.83	0.40	0.37	2.97	IVUS	0.04
8	0.60	0.42	0.01	0.47	0.30	1.15	0.49	0.45	0.96	IVUS	0.50	0.38	1.40	0.36	0.34	1.40	IVUS	0.03
9	0.42	0.17	0.12	0.35	0.14	1.26	0.32	0.29	1.30	IVUS	0.43	0.42	1.43	0.41	0.40	1.43	IVUS	0.02
10	0.46	0.16	0.01	0.35	0.25	1.04	0.26	0.23	4.47	IVUS	0.49	0.41	0.11	0.48	0.41	0.87	IVUS	0.03
11	0.53	0.20	0.33	0.49	0.19	0.87	0.37	0.33	1.24	IVUS	0.41	0.38	1.15	0.44	0.37	1.15	IVUS	0.03
12	0.49	0.18	0.01	0.34	0.27	0.92	0.37	0.35	0.89	IVUS	0.54	0.46	1.70	0.41	0.38	1.70	IVUS	0.02
13	0.36	0.16	0.01	0.28	0.21	0.40	0.33	0.32	2.35	IVUS	0.41	0.36	2.40	0.34	0.30	2.40	IVUS	0.02
14	0.56	0.32	0.01	0.66	0.41	0.56	0.57	0.55	4.04	IVUS	0.60	0.52	2.05	0.54	0.54	2.05	IVUS	0.04
15	0.47	0.17	1.20	0.43	0.25	0.54	0.46	0.43	2.37	IVUS	0.46	0.38	1.92	0.39	0.36	1.92	IVUS	0.03
16	0.42	0.39	1.56	0.33	0.28	2.47	0.39	0.36	0.53	IVUS	0.48	0.34	1.38	0.53	0.45	1.38	IVUS	0.04
17	0.11	0.45	0.11	0.12	0.39	0.80	0.24	0.26	2.70	IVUS	0.48	0.56	0.75	0.35	0.38	0.75	IVUS	0.03
18	0.41	0.05	1.37	0.27	0.35	1.37	0.34	0.37	1.67	IVUS	0.45	0.48	2.29	0.39	0.42	2.29	IVUS	0.04
19	0.49	0.25	0.20	0.36	0.28	0.53	0.32	0.28	1.99	IVUS	0.51	0.40	0.13	0.57	0.52	0.13	IVUS	0.03

Table S2. Coronary arteries. Geometrical information regarding consecutive tracts of both anomalous and normal coronary arteries are reported for each patient. In particular, the maximum axis (Max D), minimum axis (Min D), and length are shown for each tract. Measurements were taken from intravascular ultrasound (IVUS) or computational tomography (CT), indicated as a *Source* in the table. The thickness value (H) is reported as a mean value of two sides. All measurements are defined in *cm*.

	Resting		Hyperemia	
ID	RCA	LCA	RCA	LCA
1	2,163	1,002	230	219
2	864	1,429	279	268
3	508	1,503	287	386
4	1,713	1,083	409	244
5	1,473	750	448	251
6	1,373	1,058	360	206
7	935	1,611	309	275
8	1,248	1,632	297	172
9	1,610	501	341	194
10	2,098	1,356	584	567
11	4,385	1,565	770	324
12	1,517	147	437	103
13	1,304	759	597	320
14	1,749	1,347	437	271
15	549	482	385	325
16	393	444	193	207
17	960	1,097	408	351
18	449	986	224	238
19	1,860	1,956	696	663

Table S3. Coronary Boundary conditions. Coronary resistances of right and left coronary arteries in-vivo measured during catheterization exam are reported for resting and hyperemic conditions. Value are defined in *mmHg/(L/min)*.

ID	Systolic pressure	Diastolic pressure	Heart rate
1	124	82	86
2	114	72	68
3	101	69	85
4	155	77	53
5	158	83	57
6	137	78	67
7	142	73	54
8	143	75	59
9	108	69	57
10	151	67	68
11	148	90	67
12	103	76	103
13	116	58	54
14	116	75	66
15	91	58	64
16	145	64	58
17	154	90	75
18	136	74	59
19	136	83	103

Table S4. Patient data. Systolic pressure [mmHg], diastolic pressure [mmHg], and heart rate [bpm] computed as the average value of ten consecutive beats, recorded during the catheterization exam, are reported for each patient.
